# Cisplatin-Induced Nausea and Vomiting: Effect of Herbal Medicines

**DOI:** 10.3390/plants11233395

**Published:** 2022-12-06

**Authors:** Yuchan Shin, Bonglee Kim, Woojin Kim

**Affiliations:** 1 Department of Physiology, College of Korean Medicine, Kyung Hee University, Seoul 02447, Republic of Korea; 2 Korean Medicine-Based Drug Repositioning Cancer Research Center, College of Korean Medicine, Kyung Hee University, Seoul 02447, Republic of Korea

**Keywords:** cisplatin, feeding disorder, herbal medicines, nausea, vomiting

## Abstract

Cisplatin is a chemotherapeutic agent that is widely used to treat various types of cancers. However, its side effects, most commonly nausea and vomiting, limit its widespread use. Although various drugs, such as ondansetron and aprepitant, are used to alleviate these side effects, their efficacy is still debated. This review aims to summarize the results of 14 studies on the effects of seven single herbal extracts, one multiple herbal extract, and one ginger sub-component (i.e., [6]-gingerol) on cisplatin-induced nausea and vomiting. The results of the included studies were subdivided into four categories: kaolin consumption, retching and vomiting, food intake, and weight loss. Most studies used rodents, whereas four studies used minks or pigeons. The doses of cisplatin used in the studies varied from 3 mg/kg to 7.5 mg/kg, and only a single injection was used. Nine studies analyzed the mechanisms of action of herbal medicines and assessed the involvement of neurotransmitters, cytokines, enzymes, and various hematological parameters. Although further research is needed, this review suggests herbal medicine as a viable treatment option for cisplatin-induced neuropathic pain.

## 1. Introduction

Cisplatin is a widely used metal-based chemotherapeutic drug, and nearly 50% of platinum-based anticancer agents sold worldwide are cisplatin [1,2]. It is generally used for the treatment of solid cancers, such as testicular, ovarian, and gastric cancers [3]. This second-generation chemotherapeutic drug exerts anticancer effects by inhibiting cell division by blocking DNA, mRNA, and protein production [4,5,6]. With this mechanism, cisplatin effectively decreases tumor size. However, it can also induce several side effects, such as nephrotoxicity, hepatotoxicity, and gastrointestinal toxicity [3,7,8,9]. Nephrotoxicity is induced by accumulated cisplatin in tubular epithelial cells of the kidney, which leads to tubular cell injury or death [10,11]. Hepatotoxicity occurs when cisplatin is metabolized in the liver [12]. Cisplatin can also induce gastrointestinal toxicities, diarrhea, loss of taste, pancreatitis, and mucositis [13]. However, among these various gastrointestinal problems, nausea and vomiting are the most commonly induced by chemotherapeutic drugs such as cisplatin, with approximately 40% of treated patients complaining of these symptoms [14]. Nausea and vomiting in chemotherapy-treated patients can cause dehydration and undernourishment, which decrease the quality of life of patients if not appropriately controlled [15,16]. Moreover, it can cause discontinuation or reduced efficacy of the treatment [17,18,19]. Overall, these findings indicate the importance of controlling cisplatin-induced nausea and vomiting (CINV). CINV can be categorized as acute or delayed. Acute CINV occurs within the first 24 h after chemotherapy and is generally treated using serotonin (5-HT) receptor antagonists. Ondansetron suppresses the activation of 5-HT receptors located peripherally in the intestine and centrally in the area postrema [20]. Delayed CINV mostly occurs 24–120 h after chemotherapy and is related to the neurokinin 1 (NK1) receptor and pro-inflammatory cytokines [21,22]. NK1 receptor antagonists, such as aprepitant, are used to treat delayed CINV because they block the binding of substance P to the NK1 receptor [20]. In the area postrema, dopamine plays an important role, as dopamine 2-like receptors interact with the emetic receptors present in the area postrema and activate the nucleus of the solitary tract [23]. In line with this, the National Comprehensive Cancer Network has listed dopamine receptor antagonists, such as metoclopramide, as alternatives to treat emesis [20]. However, all the above-mentioned drugs can induce side effects that could limit their widespread use. Ondansetron can affect the electrical activity of the heart and cause abnormal cardiac rhythm [24]. In a study involving 1175 patients treated with 5-HT_3_ antagonists, such as ondansetron, dolasetron, and granisetron, 27.5% of the patients experienced adverse events, such as constipation (9.2%), headache (7.4%), and alanine aminotransferase (ALT) increase (3.1%) [25]. Furthermore, in a study involving 521 patients (260 patients received aprepitant, and 261 patients received standard therapy), 14.6% of the patients experienced drug-related adverse events, such as asthenia/fatigue (17.2%), hiccups (13.8%), and constipation (8.0%) [26]. Finally, metoclopramide treatment has also been associated with side effects, such as headache, dizziness, and extrapyramidal reactions [27]. Thus, efforts to identify novel drugs with limited side effects are urgently required.

Herbal medicine, which has a lower incidence of side effects than chemical drugs [28,29,30], has been used to treat gastrointestinal disorders [31,32], nausea, and vomiting for several decades [33]. For example, ginger (*Zingiberis rhizoma*) has been used for a long time to alleviate gastrointestinal discomfort, such as nausea [34], and Xiao-ban-xia-tang (XBXT) composed of *Pinelliae tuber* and *Zingiberis rhizoma* has been used for emesis [34]. In addition, the underlying mechanisms of action of some herbal medicines have been demonstrated. For example, gingerol, which is a component of *Zingiberis rhizoma*, decreases emesis by blocking 5-HT and NK_1_ receptors [35].

This review aims to summarize the results of 14 studies on the effects of herbal medicine extracts or their sub-components on CINV. Only studies conducted on animals were included to elucidate the underlying mechanisms of action of each herbal medicine. The components of the herbal extracts and their mechanisms of action were also discussed.

## 2. Results

This review included 14 studies (Table 1) on the effects of various herbal medicines on cisplatin-induced gastrointestinal complications. The results of the included studies were subdivided into four categories: kaolin consumption [34,35,36,37,38,39,40,41,42,43,44], retching and vomiting [43,45,46,47], food intake [36,37,38,39,40,41], and weight loss [34,39,40,41,42,44,47].

### 2.1. Kaolin Consumption

Kaolin is a type of clay that does not contain nutrients [48]; however, its consumption is usually used as a proxy for vomiting in rodents because this species cannot vomit [49]. Although rats have brain stem nuclei similar to those of humans, no motor component exists for vomiting [50]. Therefore, instead of vomiting, kaolin consumption is considered a sign of vomiting [51]. In total, 11 studies observed kaolin consumption in rats to assess the antiemetic effects of various herbal medicine extracts. The doses of cisplatin used were either 3 mg/kg [36,37,38,39] or 6 mg/kg [35,40,41,42,44]. Seven herbal medicines were used, all of which were administered orally [34,35,36,37,38,39,40,41,42,43,44]. These studies used male Wistar or Sprague–Dawley rats.

Aung et al. [39] assessed the effects of Scutellariae Radix (SR) on cisplatin-induced kaolin consumption in rats. Three different doses of cisplatin (3, 5, and 10 mg/kg) were intraperitoneally injected, and the lowest dose showed the strongest effect, as indicated by the continuous increase in kaolin consumption until 120 h compared with the baseline. SR was administered intraperitoneally at three different doses (1, 3, and 10 mg/kg). Results showed that pre-treatment with 1 and 3 mg/kg SR significantly reduced kaolin consumption compared with the baseline only at 24 h, whereas 10 mg/kg was effective at 24, 48, and 96 h [39]. The ratio of food intake and body weight was also measured, and only the 3 mg/kg SR-treated group showed a significant increase in kaolin consumption at 72 h. Similar to Aung et al. [39], Mehendale et al. [38] also investigated the effects of SR and observed that 3 mg/kg but not 10 mg/kg SR significantly decreased kaolin consumption after injecting 3 mg/kg cisplatin. In addition, they analyzed the effects of berries of Panacis Quinquefolii Radix (BPQ; American ginseng berry), which exerts antioxidant effects similar to those of SR. They found that intraperitoneal treatment with 50 mg/kg but not 10 mg/kg BPQ can attenuate kaolin consumption after injecting 3 mg/kg cisplatin. In their second study, Mehendale et al. [37] used higher doses of BPQ (50, 100, and 150 mg/kg) and concluded that 100 mg/kg is the most effective dose to relieve kaolin consumption. Furthermore, they assessed the effect of 5 mg/kg ginsenoside Re, the major component of the BPQ extract, and suggested that it contributed to the effect of BPQ.

Wang et al. [36] assessed the effects of the oriental fungus *Ganoderma lucidum* on kaolin consumption and found that treatment with 3 and 10 mg/kg *G. lucidum* can significantly decrease kaolin consumption at 24–120 h. *G. lucidum*, which contains 1.89% terpenoids and 15.8% polysaccharides, modulates immunity by increasing the percentages of CD5^+^, CD4^+^, and CD8^+^ T lymphocytes in peripheral blood lymphocytes [52].

The studies by Raghavendran et al. [40] and Sathyanath et al. [41] focused on Ginseng Radix (GR). Raghavendran et al. [40] applied GR pre-treatment at different doses before injecting cisplatin (7 mg/kg), but only 25 and 50 mg/kg attenuated kaolin consumption. However, when GR was administered after injecting cisplatin (6 mg/kg), three doses (12.5, 25, and 50 mg/kg) were effective in attenuating kaolin consumption. Sathyanath et al. [41] found that treatment with 6 mg/kg cisplatin can increase kaolin consumption. Although pre-treatment and post-treatment with steamed GR, also known as red ginseng, at different doses (i.e., 25, 50, and 100 mg/kg, p.o.) did not show any significant effect on kaolin consumption at 120 h, post-treatment with 50 and 100 mg/kg steamed GR significantly decreased kaolin consumption at 48 h compared with cisplatin alone. The components of GR can be divided into two categories: saponins and non-saponins. GR has relatively high concentrations of ginsenosides (GS), such as ginsenoside Rb_1_ and Rg_3_, whereas non-saponin ginsenosides (GNS) contain undetectable amounts of GS. The results indicate that post-treatment with GS and GNS orally can significantly decrease kaolin consumption at 24 h compared with cisplatin alone.

Tian et al. [43] and Cheng et al. [35] assessed the effects of gingerol after treatment with 3 and 6 mg/kg cisplatin, respectively. Tian et al. [43] found that oral treatment with 10, 20, and 40 mg/kg [6]-gingerol for three consecutive days can significantly attenuate kaolin consumption in rats up to 72 h after cisplatin treatment. Cheng et al. [35] reported that administering 50 and 100 mg/kg [6]-gingerol can significantly ameliorate cisplatin-induced kaolin consumption at 24 h [35].

Gingerol is one of the main components of *Zingiberis rhizoma*. *Zingiberis rhizoma* is a herbal medicine that has been widely used alone or in combination with other herbs. Among these, XBXT is widely used to treat various digestive problems. In this study, Meng et al. [34] and Li et al. [44] focused on the effects of XBXT on CINV.

In the study by Meng et al., kaolin consumption increased after treatment with 6 mg/kg cisplatin, but pre-treatment with XBXT 1.3 g/kg significantly decreased kaolin consumption compared with cisplatin alone at 24 and 72 h [34]. Similarly, Li et al. [44] showed that treatment with 6 mg/kg cisplatin dramatically increased kaolin consumption and that 1.6 g/kg XBXT significantly decreased kaolin consumption at 24–72 h. In another study, Meng et al. reported that oral treatment with 1.75 g/kg Forsythiae Fructus can significantly decrease kaolin consumption at 0–48 h in 6 mg/kg cisplatin-injected rodents [42].

### 2.2. Retching and Vomiting

Three studies used mink [43,45,46] and one used pigeon [47] to assess the effects of herbal medicines on CINV. Unlike rodents, minks and pigeons retch and vomit [53]. In minks, increased 5-HT from enterochromaffin cells of the intestine play an important role in humans [54].

Qian et al. [46] assessed the effects of gingerol on CINV in minks. Three doses (50, 100, and 200 mg/kg) of [6]-gingerol were intraperitoneally administered before injecting 7.5 mg/kg cisplatin. In the cisplatin-injected group, the numbers of retching and vomiting episodes for 6 h were 70.83 ± 16.49 and 9.67 ± 2.58, respectively. However, [6]-gingerol pre-treatment significantly attenuated these effects. In the 200 mg/kg-treated group, the effect was stronger as the numbers of retching and vomiting episodes decreased to 5.50 ± 13.47 and 0.84 ± 2.04, respectively. In the other study conducted by Qian et al. [45], the effect of XBXT on cisplatin-injected minks was assessed. Two doses of XBXT were injected intraperitoneally, and the results showed that they dose-dependently attenuated CINV for 72 h.

Tian et al. [43] used minks to assess the effects of [6]-gingerol. The doses used were also similar to those used by Qian et al. [46]; however, they administered gingerol orally and not intraperitoneally. The results showed that 100 and 200 mg/kg gingerol significantly decreased vomiting after 72 h, whereas the effect of 50 mg/kg gingerol diminished after 42 h. The anti-retching effect of gingerol did not last until 72 h in all three groups, and the significant differences disappeared before 72 h.

In contrast to the above-mentioned studies, Ullah et al. [47] used pigeons to observe changes in retching and vomiting after treatment with cisplatin and *B. monnieri*. They used methanol and n-butanol for extraction. The n-butanolic fractions of *Bacopa monnieri* are rich in bacoside. Their results showed that the methanolic (10–40 mg/kg) and bacoside-rich n-butanolic fractions of *B. monnieri* (5–20 mg/kg) attenuated cisplatin-induced emesis by 66.3% and 71.6%, respectively, whereas the widely used standard antiemetic drug metoclopramide (30 mg/kg) produced only a 48.9% reduction.

### 2.3. Food Intake

Decreased food intake following cisplatin injection has been reported in several studies [55]. Although the relationship of nausea and vomiting with decreased food intake is not clearly understood, cisplatin reportedly decreases food intake by inhibiting intestinal motility [56]. It induces morphological changes in the small intestine by lowering the area occupied by the microvilli and villi. Moreover, inflammation in the mucosal and submucosal layers has been shown [57]. In the present review, nine studies focused on the changes in food intake after treatment with cisplatin at 3 mg/kg [36,37,38,39] or 6 mg/kg [34,35,40,41,42,44] and seven herbal medicines. Three herbal medicines were administered intraperitoneally [37,38,39], and the others were administered orally [34,35,36,37,38,39,40,41,42,44].

In the studies by Aung et al. [39] and Wang et al. [36], 3 mg/kg cisplatin decreased food intake in rats. Aung et al. analyzed food intake by measuring the food intake: body weight ratio, whereas Wang et al. measured the 24 h food intake. In the study by Aung et al., 3 mg/kg SR significantly increased food intake at 72 h compared with cisplatin. Meanwhile, Wang et al. found that administration of an *G. lucidum* extract can dose-dependently (1, 3, and 10 mg/kg) increase food intake in rats.

Mehendale et al. [37] recorded the food intake percentage compared with the baseline. In the cisplatin group, food intake decreased to 57% of baseline at 24 h, whereas 50 and 100 mg/kg BPQ increased food intake to 76% and 85% of baseline at 24 h. However, 150 mg/kg BPQ failed to show any statistically significant differences compared with the control. Raghavendran et al. [40] and Sathyanath et al. [41] assessed the effects of GR on food consumption. Raghavendran et al. found that multiple pre-treatments with two low doses of GR (25 and 50 mg/kg, i.p.) increased food intake until 120 h, whereas pre-treatment with 100 mg/kg GR was ineffective. In multiple post-treatments with GR, only the lowest dose increased food intake until 72 h. In the study by Sathyanath et al., the steamed root of GR failed to affect food intake, and all three doses (25, 50, and 100 mg/kg) did not affect food intake in rats. However, pre-treatment with 10 and 100 mg/kg GS and GNS, respectively, increased food intake in rats. In a study by Meng et al. [42], Forsythiae Fructus significantly increased food intake compared with cisplatin only at 48 h but not at 24 and 72 h after cisplatin injection.

In contrast to other herbal extracts, XBXT treatment failed to increase food intake in cisplatin-injected rodents [34,44].

### 2.4. Body Weight

Cisplatin-induced gastrointestinal toxicity can decrease food intake and body weight. Eight studies reported changes in body weight after treatment. The doses of cisplatin used were 3 mg/kg [39], 6 mg/kg [34,35,40,41,42,44], and 7 mg/kg [47]. Six herbal medicines were used in the present study. One herbal medicine was administered intraperitoneally [39], and the others were administered orally [34,35,39,40,41,42,44,47].

In the study by Aung et al. [39] all three doses of SR failed to increase body weight, which was attenuated after cisplatin injection.

GR was assessed by Raghavendran et al. [40] and Sathyanath et al. [41]. In the study by Raghavendran et al. [40], pre- and post-treatment with GR significantly increased the body weights of cisplatin-treated rats. However, the effect was stronger in pre-treated rats than in post-treated rats. Specifically, pre-treatment with 25 and 50 mg/kg GR induced significant changes at 96 h after cisplatin injection, whereas post-treatment with 12.5 and 25 mg/kg GR induced changes at 48 h. In a study conducted by Sathyanath et al. [41], no significant differences were observed between rats treated with the extract of steamed GR and rats treated with its subcomponents GS and GNS. In the study by Ullah et al. [47] methanolic fractions of *B. monnieri* significantly increased body weight compared with cisplatin alone; the mean weight loss was 15.3% ± 1.4% in the cisplatin group, whereas the loss decreased to 8.3% ± 1.6%, 5.2% ± 1.0%, and 5.6% ± 1.6% in the groups treated with 5, 10, and 20 mg/kg *B. monnieri*, respectively. Similar to their effects on food intake, XBXT [34] and [6]-gingerol [35] failed to prevent or diminish body weight loss induced by cisplatin injection.

### 2.5. Herbal Medicines Used in the Studies

This review also discussed the effects of eight types of herbal medicine extracts or their components, including seven single herbal extracts [35,36,37,38,39,40,41,42,43,45,46,47], one multiple herbal extract (i.e., XBXT) [34,44,45], and one component of *Zingiberis rhizoma* (i.e., [6]-gingerol), on CINV. Furthermore, eight studies [34,36,37,38,39,40,41,42,47] used high-performance liquid chromatography to identify and quantify the components in their herbal extracts (Table 2).

### 2.6. Mechanisms of Action

In this review, nine studies elucidated the underlying mechanisms of action of herbal extracts (Table 3). Four studies [35,43,46,47] investigated neurotransmitters, particularly 5-HT and dopamine. Two studies [43,46] investigated substance P. Three studies [35,43,44] analyzed the effects of tryptophan hydroxylases (TPHs), which are enzymes engaged in 5-HT formation. 5-HT has been reported to play an important role in nausea and vomiting as a chemotherapeutic agent was shown to stimulate the EC cells of the gastrointestinal tract mucosa and induce a calcium-dependent 5-HT release [58]. The released 5-HT is known to affect the vagal afferent nerve and stimulate the vomiting center in the dorsal vagal center located in the brain [59]. The nucleus of the solitary tract (NTS) of the dorsal vagal center sends signals to the dorsal motor of the nucleus of the vagus nerve, which in turn mediates the emetic motor function of the gastrointestinal tract in the process of vomiting [60].

Two studies [34,42] investigated the impact of pro-inflammatory cytokines, such as interleukin (IL)-1 and IL-8, and substances that induce the formation and activation of cytokines, such as NLRP3, ASC, and Caspase-1. Pro-inflammatory cytokines are reported to affect the vagal afferent nerves endings and convey information to the NTS, as the vagus nerve is reported to be sensitive to peripheral pro-inflammatory cytokines, such as TNF-α, IL1-β, and IL-6 released by macrophages [61,62].

One study [40] reported their effects on white blood cells, such as leukocytes, hemoglobin, and NK cells. One study [45] reported their effects on NK receptor. Five studies [34,40,41,42,44] investigated histological deformities, and three studies [34,35,42] focused on the role of oxidative stresses, such as monoamine oxidase A (MAO-A) and serum reactive oxygen species (ROS).

## 3. Discussion

This review summarized the findings of 14 studies on the effects of herbal medicine extracts or their sub-components. To our knowledge, this is the first study to assess the effects of herbal medicines on CINV. Cisplatin is a platinum-based chemotherapeutic drug widely used to treat various cancers, such as head and neck, bladder, and ovarian cancers [63]. However, it can induce serious nausea and vomiting, which can affect the quality of life of patients [15,16]. The European Society of Medical Oncology and the Multinational Association of Supportive Care in Cancer classified cisplatin as a drug with high emetic risk [64].

In the studies included in this review, cisplatin was administered intraperitoneally to animals to induce nausea and vomiting. The doses of cisplatin used varied from 3 mg/kg to 7.5 mg/kg, and only a single injection was used. As mentioned in our previous study [55], humans (60 kg) are generally injected with cisplatin at a dose of 35 mg/$m^{2}$. When cisplatin is applied to rodents, 5 mg/kg is the most appropriate dose [65], showing that the doses used in the studies were within the range. Minks [43,45,46], pigeons [47] or rats [34,35,36,37,38,39,40,41,42,43,44], all of which were males, were used in the included studies.

Among the included studies, [6]-gingerol was the most frequently investigated component, as its effect was assessed in three [35,43,46] studies. [6]-gingerol is a main component of ginger (*Zingiberis rhizoma*) along with [6]-shogaol [34] and 11% of ginger is gingerol [66]. Gingerol has many pharmacological effects, such as anti-cancer, anti-inflammatory, and gastric activity-promoting effects [66]. In the included studies, gingerol was administered orally or intraperitoneally to the rats or minks. The administered dose ranged from 10 mg/kg to 200 mg/kg in rats, whereas in minks, the lowest dose was 50 mg/kg. All treated doses significantly attenuated kaolin consumption or nausea and vomiting; however, intraperitoneal administration of 50 mg/kg gingerol in minks failed to induce significant effects after 48 h. The effect of 200 mg/kg gingerol was similar to that of 2 mg/kg ondansetron [43]. As the mechanism of action, it significantly decreased the upregulated 5-HT, dopamine, and substance P levels in the intestine (ileum) and the brain (area postrema and medulla oblongata). Future studies could investigate the effects of ginger extract and shogaols because they are similar to gingerol.

Two studies used GR. One study used an extract of GR [40], whereas the other used an extract of steamed GR [41]. Both studies assessed the pre- and post-treatment effects of GR. Their results showed that GR exerted significant effects when administered before and after treatment; however, steamed GR failed to prevent nausea and vomiting in rats. Meanwhile, post-treatment with GR decreased nausea and vomiting. This difference in effect may be ascribed to the difference in the amounts of GS present in GR. In the steamed GR, the amounts of Rb_1_ (3.93 vs. 5.14), Rb_2_ (1.92 vs. 3.60), and Rg_1_ (0.422 vs. 7.22) were smaller than those in the normal GR. Furthermore, no Rb_3_ (no vs. 6.33) was detected. Although the amount of Rg_3_ was larger in steamed GR (2.687 vs. 1.08), this difference may not have been significant. In 2019, a meta-analysis reported that ginsenoside Rg_3_ can relieve the side effects of chemotherapy against digestive system cancers, suggesting that Rg_3_ could alleviate nausea and vomiting [67]. GS are triterpenoidal glycosides with high chemical variation, and they are considered to play critical roles in the bioactivity of GR [68]. More than 30 types of GS have been isolated, and novel ginsenosides have been discovered [69].

The underlying mechanisms of action of herbal medicines were assessed in nine studies (Table 3). 5-HT was the most mentioned, as five studies [35,43,44,46,47] have investigated on its role in the effect of herbal medicines. 5-HT is involved in the development of emesis [70]. Chemotherapy drugs stimulate EC cells to secrete 5-HT [71,72] and further stimulate their receptors, which are mostly located at the vagal afferent fibers. The signals are further transmitted to the brain in the vomiting center [71], and impulses could be evoked to the emesis reflex. In four studies [35,43,46,47], cisplatin increased 5-HT levels in the postrema, medulla oblongata, and ileum of rats or minks; however, administration of gingerol decreased 5-HT levels in the brain. In addition, the level of 5-HT_3A_ significantly increased after cisplatin treatment, whereas gingerol administration significantly decreased it in the medulla oblongata and ileum [35]. Furthermore, TPH-1 and -2, which are the enzymes that initiate the formation of 5-HT [43], were upregulated after cisplatin treatment and downregulated after herbal medicine administration. MAO-A participates in 5-HT metabolism by inducing oxidation [35]. Three studies [35,43,44] reported that XBXT and [6]-gingerol can increase these factors in rats and minks. XBXT is composed of *Zingiberis rhizoma* and Pinelliae Tuber, and [6]-gingerol is one of the main components of ginger. Thus, the 5-HT modulating effect of XBXT may be due to ginger and its sub-component, [6]-gingerol. Gingerols have been reported to easily penetrate the BBB [73]. Cisplatin is known to poorly penetrate the BBB, with less than 5% of intravenously injected cisplatin found in the brain [74], and the changes in the 5-HT system in the brain after cisplatin injection remains to be studied, whether it is a direct or indirect effect of cisplatin. However, the effect of [6]-gingerol on the brain 5-HT system appears to be the direct effect, as [6]-gingerol is reported to enter the brain by passive diffusion [73]. Furthermore, gingerols have been reported to have 5-HT antagonistic effects [75,76,77]. Herbal medicines can also reduce cisplatin-induced gastric toxicity. In two studies [34,42], cisplatin increased serum ROS and pro-inflammatory cytokines, such as IL-18 and IL-1β, in the antrum and ileum of rats; however, XBXT and Forsythiae Fructus significantly decreased serum ROS and cytokine levels. Furthermore, herbal medicine extracts can restore histopathological damage, such as destruction of the epithelial cells of the gastric mucosa surface. In five studies [34,40,41,42,44], XBXT, GR, and Forsythiae fructus attenuated histopathological damage to the mucosal surface of the rat antrum and ileum. GR has been assessed by two studies, and it significantly regulated the hematological parameters and histological deformities induced by cisplatin. GR has been reported to be effective against various types of diseases. It has been used worldwide for thousands of years for treatment of cancer [78], diabetes [79], and postmenopausal symptoms [80].

In our previous study [55], we discussed the effects of herbal medicines on cisplatin-induced anorexia. Among 14 studies included in this review, three were also analyzed in the previous review [37,39,40]. However, most of the herbal medicines used were different in the two studies. Furthermore, the pathophysiological mechanisms focused on were also different in both studies; in the anorexia study, the role of feeding regulating hormones, such as ghrelin and leptin, were assessed in seven studies, whereas in this review, neuropeptides (i.e., substance P and NK receptor) and 5-HT regulating enzymes (i.e., TPH and MAO-A) were focused on by more than seven studies. 5-HT and its receptors are known to play a major role in various types of feeding disorders [81]. However, some studies have reported that different 5-HT receptors are involved in the mechanisms of anorexia and nausea and vomiting, as the role of 5-HT_2B_ and 5-HT_2C_ receptors was shown to be more critical than that of the 5-HT and 5-HT_3_ in anorexia [82,83], whereas the opposite has been reported in nausea and vomiting [70].

Although this review only focused on studies conducted in animals, the efficacy of herbal medicines has also been reported in clinical trials. In a randomized phase II study conducted on 40 cancer patients receiving chemotherapy (i.e., cisplatin and paclitaxel), rikkunshito, which is a water-extract of a mixture of eight herbal medicines, demonstrated an additive effect on the prevention of nausea and vomiting [84]. Furthermore, a double-blind and multicenter trial, which involved randomly selected 744 cancer patients undergoing chemotherapy, has reported that ginger supplementation at a daily dose of 0.5 g–1.0 g could reduce acute nausea and vomiting in adult cancer patients [85].

In conclusion, this review shows that herbal medicine extracts can be used to attenuate CINV. However, well-designed studies must be conducted in the future to draw any conclusions. The effects of diverse herbal medicines should be evaluated, and the effects of ginger, shogaols, and GS are worthy of evaluation in the future.

## 4. Materials and Methods

**Figure 1:**
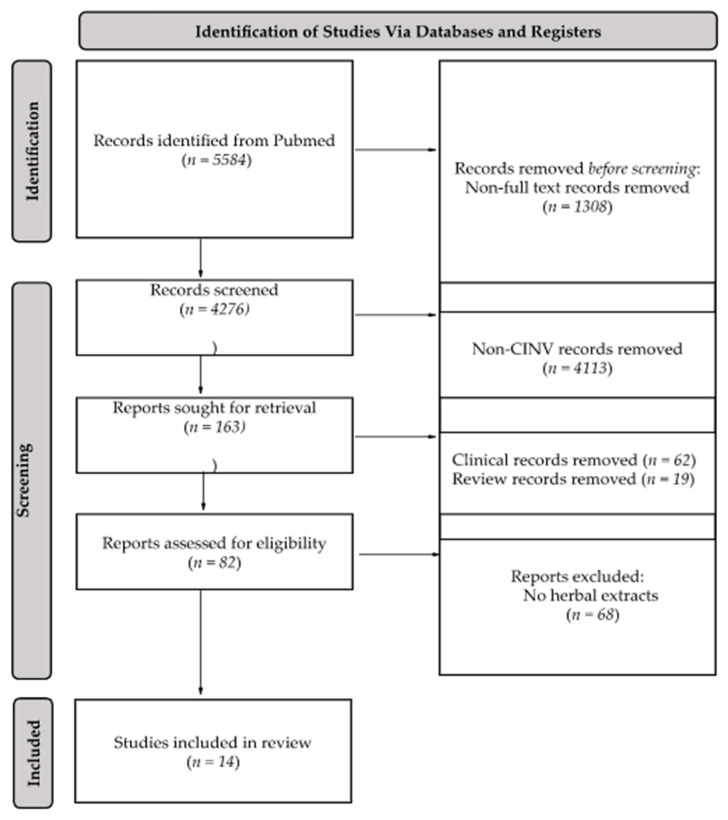
Flow chart of article inclusion protocol. In total, 5584 articles were screened by abstract and full-text examinations. Finally, a total of 14 articles assessing the effect of herbal extracts in cisplatin-induced nausea and vomiting in animals were included in our review.

A search was conducted for all studies on herbal medicines and CINV in Google Scholar and the National Library of Medicine (MEDLINE) using PubMed (Figure 1). Studies that were published electronically until the end of February 2022 and were written in English were included. The literature search was performed using the following keywords: “Chemotherapy-Induced Nausea and Vomiting”, “Cisplatin”, “Emesis”, “Herbal Medicine”, “Nausea”, and “Vomiting”. After the initial search, duplicates, bibliographies, study protocols, clinical trials, and non-English language studies were excluded. Fourteen studies were included in the present review.

## Figures and Tables

**Table 1:** Summary of Studies on Cisplatin Induced-Nausea & Vomiting.

Herbal Medicine	Doses	Animals	Cisplatin	Kaolin Intake	Retching & Vomiting	Food Intake	Body Weight	Mechanism of Action	Authors
Xian-Bao- Xia-Tang (XBXT)	1 & 4 g/kg (i.p.)	Male Mink	Single 6 mg/kg (i.p.)	-	↑	-	-	NK1R: ↓	Qian et al., 2010 [45]
	1.6 g/kg (p.o.)	Male Wistar Rat	Single 6 mg/kg (i.p.)	↓	-	NS	NS	Ros, IL-1β, IL-18, NLRP3, Caspase-1: ↓	Meng et al., 2020 [34]
	1.6 g/kg (p.o.)	Male Wistar Rat	Single 6 mg/kg (i.p.)	↓	-	NS	NS	CD86, TNF, TRPV2, Map3k8, NLRP3 IL-1R1, IL-1β, IL-6, IL-34: ↓	Li et al., 2020 [44]
[6]-gingerol	50, 100, 200 mg/kg (p.o.)	Male Mink	Single 7.5 mg/kg (i.p.)	-	↓	-	-	5-HT, DA, Substance P: ↓	Qian et al., 2009 [46]
	10, 20, 40 mg/kg (rat) & 50, 100, 200 mg/kg (mink) (p.o.)	Male Wistar Rat & Mink	Single 3 mg/kg (i.p., rat), 6 mg/kg (i.p., mink)	↓	↓	-	-	5-HT, 5-HT_3_, TPH-1, -2, SP, NK1R, DA, D2R, TH: ↓	Tian et al., 2020 [43]
	50 & 100 mg/kg (p.o.)	Male SD Rat	Single 6 mg/kg (i.p.)	↓	-	NS	NS	5-HT, 5-HT_3A_, TPH-1, -2: ↓ MAO-A: ↑	Cheng et al., 2020 [35]
Ginseng Radix (GR)	Pre-treatment: 25, 50, 100 mg/kg Post-treatment: 12.5, 25, 50 mg/kg (p.o.)	Male SD Rat	Single 6 or 7 mg/kg (i.p.)	↓	-	↑	↑	Pre-: Neutrophil, Lymphocytes, WBC: ↓ Post-: Hemoglobin, RBC: ↓	Raghavendran et al., 2010 [40]
	Steamed GR: 25, 50, 100 mg/kg GS: 5, 10 mg/kg GNS: 50, 100 mg/kg (p.o.)	Male SD Rats	Single 6 mg/kg (i.p.)	↓	-	NS	↑	Deformity (stomach, small intestine) 25 mg/kg: ↓ 50 & 100 mg/kg: NS	Sathyanath et al., 2013 [41]
Scutellariae Radix (SR)	1, 3, 10 mg/kg (i.p.)	Male Wistar Rat	Single 3, 5, 10 mg/kg (i.p.)	↓	-	↑	NS	-	Aung et al., 2004 [39]
	3, 10 mg/kg (i.p.)	Male Wistar Rat	Single 3 mg/kg (i.p.)	↓	-	-	-	-	Mehendale et al., 2004 [38]
Berry of Panacis Quinquefolii Radix (BPQ)	10, 50 mg/kg (i.p.)	Male Wistar Rat	Single 3 mg/kg (i.p.)	↓	-	-	-	-	Mehendale et al., 2004 [38]
	50, 100, 150 mg/kg (i.p.)	Male Wistar Rat	Single 3 mg/kg (i.p.)	↓	-	↑	-	-	Mehendale et al., 2005 [37]
Ganoderma Lucidum (GL)	1, 3, 10 mg/kg (i.p.)	Male Wistar Rat	Single 3 mg/kg (i.p.)	↓	-	↑	-	-	Wang et al., 2005 [36]
*Bacopa* *monnieri* (BM)	N-butanolic Fraction: 10, 20, 40 mg/kg & Methanolic Fraction: 5, 10, 20 mg/kg (i.m.)	Male & Female Pigeon	Single 7 mg/kg (i.v.)	-	↓	-	↑	NA: NS 5-HT, DA: ↓	Ullah et al., 2014 [47]
				-	↓	-	NS		
Forsythiae Fructus (FF)	1.7 g/kg (p.o.)	Male Wistar Rat	Single 6 mg/kg (i.p.)	-	↓	-	NS	Ros, IL-1β, IL-18, NLRP3, Caspase-1: ↓	Meng et al., 2021 [42]

Abbreviations: ↑, Increase; ↓, Decrease; 5-HT, Serotonin; D2R, Dopamine D2 Receptor; DA, Dopamine; GS, Saponin; GNS, Non-Sponin; I.g, Intragastric; IL, Interleukin; I.m., Intramuscular; I.p., Intraperitoneal; NK1R, Neurokinin-1 Receptor; MPG, N-(2-mercaptopropionyl) Glycine; NA, Noradrenaline; NLRP3, NLR Family Pyrin Domain Containing 3; NS, Nonsignificant; P.o., Per Os; RBC, Red Blood Cell; ROS, Reactive Oxygen Species; TNF, Tumor Necrosis Factor; TPH, Tryptophan Hydroxylase; TRPV2, Transient Receptor Potential Vanilloid Family Type 2; WBC, White Blood Cell.

**Table 2:** Identified and Quantified Sub-Components of Herbal Medicines Used in Studies.

Herbal Medicine/ Collected Locations	Preparation (Extraction)	Components
*Bacopa monnieri*/ Pakistan [47]	(Not Mentioned) Methanol	Bacoside A_3_, Bacopaside Ⅱ, Bacopsaponin C
Berry of Panacis Quinquefolii Radix/ United States [37,38]	75% Ethanol	Protopanaxadiol Ginsenoside: Rb_1_, Rb_2_, Rc, Rd Protopanaxatriol Ginsenoside: Re, Rg1
Forsythiae Fructus/ China [42]	100% Water	Forsythiaside A (2.62%), Forsythin (0.28%)
*Ganoderma lucidum*/ China [36]	5% Ethanol	Terpenoids (1.89%): Ganoderic acid A, Ganoderic acid C2, Ganodermanontriol Polysaccharides (15.8%)
Ginseng Radix/ Korea [40]	100% Water	Protopanaxadiol Ginsenoside: Rb_1_ (5.14 mg/g of GR), Rb_2_ (3.60), Rb_3_ (6.33), Rc (2.61), Rd (0.43), Rg_3_ (1.08) Protopanaxatriol Ginsenoside: Re (2.21), Rg_1_ (7.22), Rg_2_ (0.67), Rh_1_ (0.58), Rh_2_ (0.02)
Scutellariae Radix/ China [38,39]	100% Water	Wogonin (51.5%), Baicalein (35.6%), Skullcapflavon Ⅰ (4.8%), Skullcapflavon Ⅱ (8.3%)
Steamed Ginseng Radix/Korea [41]	100% Water	Protopanaxadiol Ginsenoside: Rb_1_ (3.93 mg/g of GR), Rb_2_ (1.92), Rc (2.04), Rd (1.07), Rg_3_ (2.68) Protopanaxatriol Ginsenoside: Rf (0.97), Re (0.74), Rg_1_ (0.42), Rg_2_ (1.58), Rh_1_ (0.91)
XBXT (Pinelliae Tuber 2: *Zingiberis rhizoma* 1)/China [34,44,45]	100% Water	Pinelliae Tuber: Ephedrine (0.309 mg/g of XBXT), Succinic acid (0.025) *Zingiberis rhizoma*: [6]-gingerol (0.0616), [6]-shogaol (0.0025)

**Table 3:** Mechanisms of Action of Herbal Medicines.

Indicator		Cisplatin	Herbal Medicines		Location
Neurotransmitters	5-HT/5-HT_3_R	↑	[6]-gingerol [35,43,46]	↓	Area postrema, Medulla oblongata, Ileum
	DA		BM [47]		Area postrema, Brain stem, Small Intestine
	NA	NS		NS	
Neuropeptide	Substance P	↑	[6]-gingerol [43,46]	↓	Area Postrema, Ileum
	NK_1_ Receptor		XBXT [45]	↓	Ileum, Area Postrema
Enzymes	TPH-1, -2	↑	[6]-gingerol [35,43,44]	↓	Medulla Oblongata, Ileum
	MAO-A	↓		↑	Medulla Oblongata, Ileum
	Caspase-1	↑	FAE [42]	↓	Antrum, Ileum
Cytokine	IL -1R1, -1β, 6, -18, -34,	↑	XBXT [34]	↓	Serum
	NLRP3				Antrum, Ileum
			FAE [42]		
Hematological Parameters	Hemoglobin	↑	GR [40]	NS	Serum
	Lymphocytes			↓	
	Monocytes			NS	
	Neutrophils			↓	
	RBC			NS	
	WBC			↓	
Histological Deformity	-	↑	XBXT [34,44]	↓	Antrum, Ileum
	-		GR [40,41]		Stomach, Small Intestine
	-		FAE [42]		Antrum, Ileum
Oxidative Stress	ROS	↑	XBXT [34]	↓	Serum, Antrum, Ileum
			FAE [42]		

Abbreviations: ↑, Increase; ↓, Decrease; 5-HT, Serotonin; 5-HT_3_R, 5-HT_3_ Receptor, BM, *Bacopa monnieri*; DA, Dopamine; FAE, Forsythiae Fructus; GR, Ginseng Radix; IL, Interleukin; MAO-A, Monoamine oxidase A; NA, Noradrenaline; NK Cell, Natural Killer Cell; NLRP3, NLR family pyrin domain containing 3; NS, Non-Significant; XBXT, Xian-Bao-Xia-Tang; ROS, Reactive Oxygen Species; TPH, Tryptophan Hydroxylase; WBC, White Blood Cell.

## Data Availability

Not applicable.
